# The Pivotal Role of Imaging in TAVR Procedures

**DOI:** 10.1007/s11886-018-0949-z

**Published:** 2018-02-12

**Authors:** Caroline Bleakley, Mark J. Monaghan

**Affiliations:** Kings College Hospital, Denmark Hill, London, SE5 9RS UK

**Keywords:** 3D transoesophageal echo, Aortic stenosis, Transcatheter aortic valve implantation

## Abstract

**Purpose of Review:**

Transcatheter aortic valve replacement (TAVR) is underpinned by an array of imaging techniques designed to not only select an appropriately sized implant but also to identify potential obstacles to procedural success. This review presents currently important aspects of TAVR imaging, describing the salient features of each modality as well as recent developments in the field.

**Recent Findings:**

The latest data on TAVR outcomes reflects the increasing experience of operators and the significant role of pre-procedural imaging. Debate continues as to which modality sizes the aortic annulus most accurately, 3D transoesophageal echocardiography (TEE) or MDCT, as well as to whether the merits of real-time peri-procedural 3D imaging guidance outweigh the possible adverse consequences of general anaesthesia which is requisite for intraprocedural 3D TEE.

**Summary:**

TAVR is now largely based on pre-acquired roadmaps of the truncal vasculature and intense pre-procedural planning. TEE and Multi-detector computed tomography (MDCT) have been shown to perform similarly in annulus sizing. However, given the complexity of many TAVR patients and the importance of identifying the most suitable pathway to the valve as well as any potentially confounding other structural or functional heart disease, both modalities remain relevant in current TAVR.

## Introduction

Transcatheter aortic valve replacement (TAVR) is now a well-established alternative to surgical aortic valve replacement (SAVR), offering the opportunity of treatment of severe aortic stenosis to those at prohibitive surgical risk. Performed without direct visualisation of the valve, TAVR is reliant on non-invasive imaging both to guide implant selection and to create a virtual roadmap of the truncal vasculature which acts as the access pathway. With fewer procedures now performed under transoesophageal echocardiography (TEE) guidance, pre-procedural imaging must be meticulous in order to minimise avoidable harm. This review will present currently important aspects of TAVR imaging, describing the salient features of each modality as well as recent developments in the field.

## The Aortic Annulus and Its Changing Profile

**Fig. 1:**
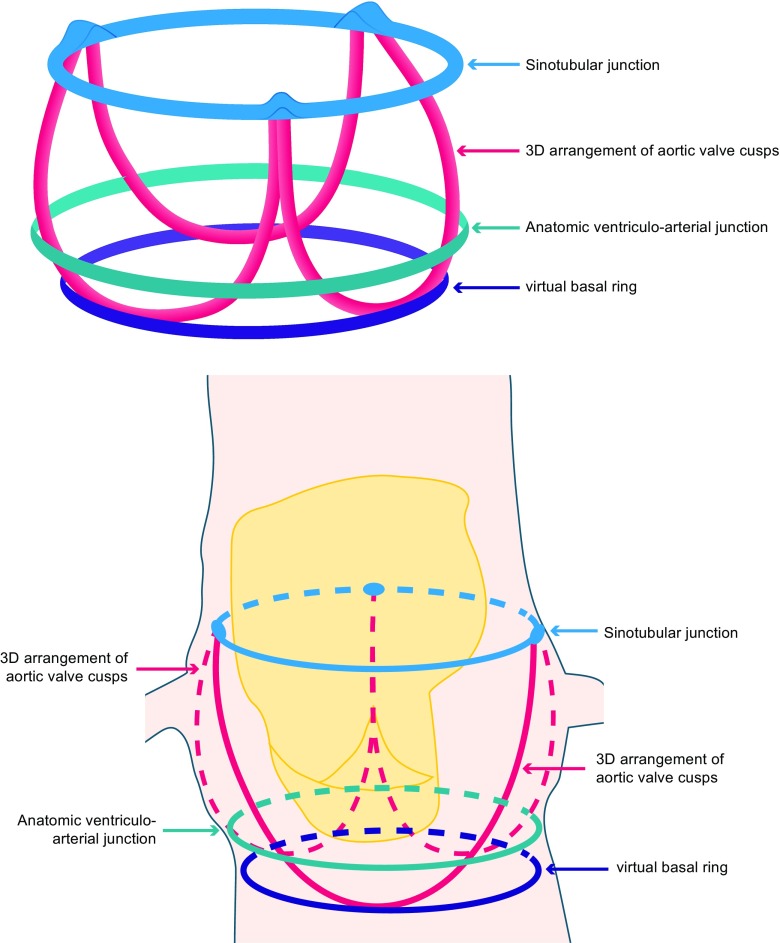
Graphical depiction of the aortic annulus. The virtual ring formed by the basal hinge points of the valve cusps is the measured annulus in TAVR sizing

In order to understand some of the challenges involved in TAVR imaging, the complexity of the aortic apparatus should be appreciated. The annulus referred to in all TAVR measurements is not actually an anatomic one but rather a virtual entity composed of a ring formed by the basal hinge points of the valve cusps [1] (Fig. 1). Therefore, this is essentially a geometric best-fit of what an anatomic annulus would look like in this position and is actually ovoid in most cases rather than circular [2•]. This has implications for its measurement, particularly diameters obtained by 2D echocardiography, as the sagittal plane that is used to obtain the left ventricular outflow tract (LVOT) diameter represents the smallest cross-section with the longest length lying in the coronal plane. Indeed, in progressively severe aortic stenosis the annulus becomes increasingly elliptical as a result of remodelling in the LVOT [3], making the use of 2D echocardiography outdated in this area with almost unavoidable underestimation of annular size.

In addition to accounting for its elliptical profile, it is important to be aware of the change in annular measurements that occur across the cardiac cycle. TEE measurements are obtained during mid-systole when the annulus is at its largest and most circular. This is the preferred time point to size the annulus, as TAVR implants predominantly assume a circular shape after deployment [4]. Conversely, MDCT measurements size the annular area at any point in the cardiac cycle depending on where the optimal image is obtained. The issue of timing in relation to measurements obtained by both TEE and MDCT was recently studied in a trial that found the best correlation to be between systolic TEE and diastolic MDCT measurements [5], whereas comparative systolic measures returned MDCT oversizing by approximately 0.28 mm^2^. It must be noted, however, that discrepant time point sizing between modalities has not been proven to be a source of significant error in device selection [6].

## Perimeter or Area

Having an appreciation of the anatomy of the aortic valve, its elliptical profile and its potential to change geometrically during the cardiac cycle, it is then interesting to consider the impact of these characteristics on annular sizing. Recent sizing charts for commercially available implantable valves are based on 3D area-derived measurements. There is, however, debate as to whether perimeter is actually the more appropriate measure. While good correlation has been demonstrated between area and perimeter measures with seemingly equal predictive power for paravalvular regurgitation [2•, 7], there are logical advantages in adopting the latter. As the annulus becomes progressively oval, area measurements are subject to potentially greater error as the area reduces in size disproportionately to the perimeter leading to potential underestimation of the true annular dimensions, an important potential source of unintentional undersizing [8]. Our group recently published a study of 262 patients comparing area- and perimeter-derived aortic valve sizing methods, with a different prosthesis selected in 26.7% of cases depending on which method was used [9]. The authors of this review are of the opinion that perimeter sizing is a more robust measure, being less susceptible to inappropriate underestimation in the context of annular elongation.

## Pre-Procedural Imaging

Accurate sizing of the intended aortic implant is central to the success of TAVR and is the source of some debate surrounding pre-procedural imaging in these patients. As stated previously, there are inherent technical issues with the use of 2D TEE, with limited ability either to account for the elliptical annulus or to identify the left main stem (LMS) origin which lies in the coronal plane. 3D TEE on the other hand is not subject to these limitations and can measure the distance from annulus to LMS origin as well as determine the length of the left coronary cusp which has the potential to occlude the ostium following valve deployment.

**Fig. 2:**
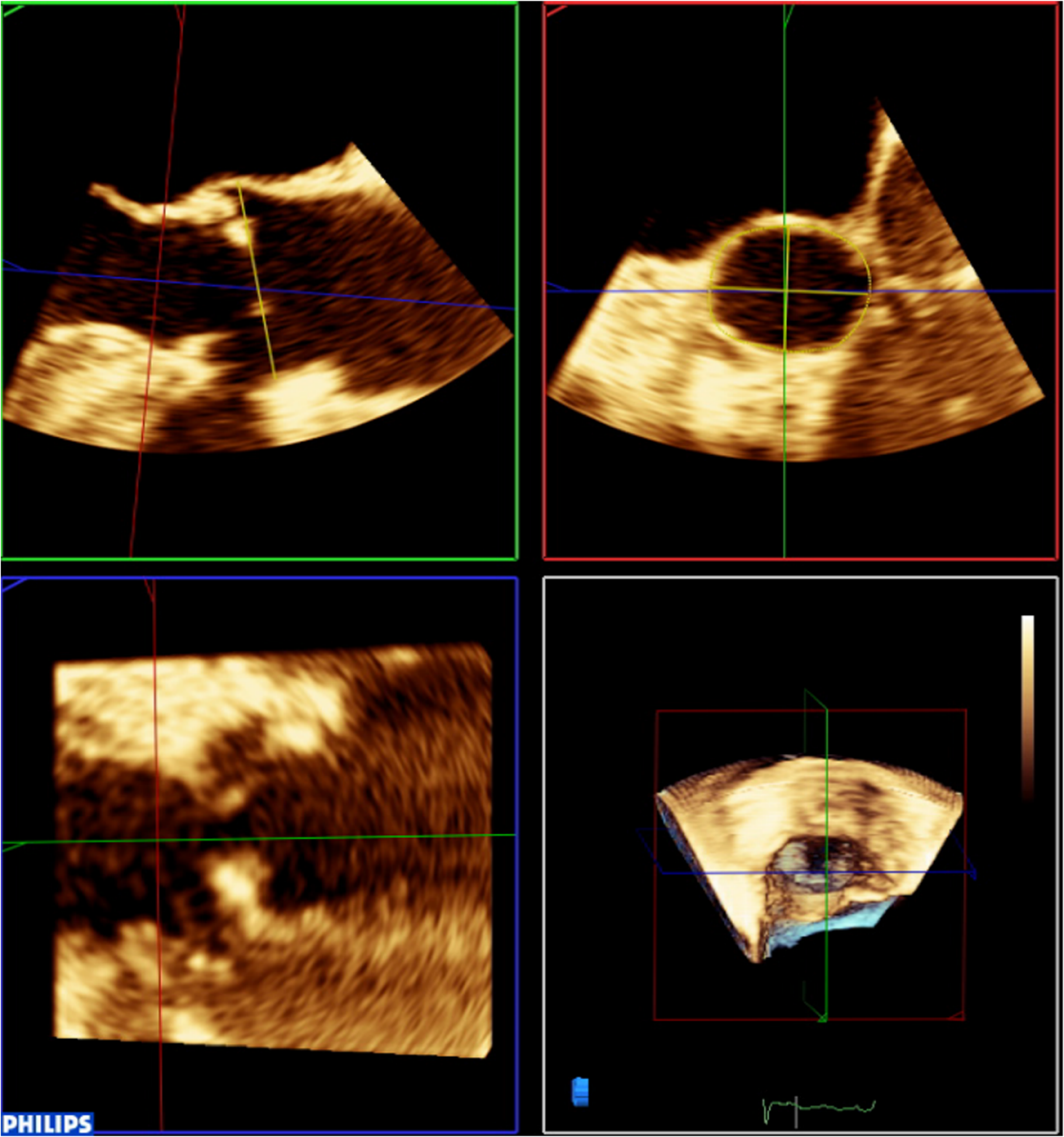
Example of 3D TEE annular sizing. The *yellow lines* drawn by the operator measure the annulus and sinus of Valsalva dimensions. Upper left panel = sagittal view; upper right panel = transverse view; bottom left panel = coronal view

With recent improvements in 3D echo technology and software, the use of 2D imaging has been largely superseded by 3D (Fig. 2). Concurrently, MDCT has evolved as a now well-validated tool, often cited as the gold standard in annular measurements. However, MDCT and 3D TEE have been shown to perform similarly in annular sizing. Several studies report good correlation between modalities with both methods having equivalence in predicting moderate or severe paravalvular regurgitation, an important potential complication of transcatheter heart valve (THV) implantation [10–12]. In a recent small retrospective study [13], TEE, MDCT and MRI all performed comparatively well with surgical device sizing, while a larger trial demonstrated good concordance in measurements for both area and perimeter between the three modalities [7].

In spite of this, there has been concern that 3D TEE tends to undersize relative to MDCT with figures between 9 and 12% quoted for the degree of discrepancy [14–16]. Prosthesis sizing is of critical importance in TAVR; if the device is undersized, there is risk of significant paravalvular leak or device embolisation. However, if the valve is oversized, the implant may disrupt the aortic root leading to rupture or, more frequently, bundle branch block or complete heart block due to impingement on the neighbouring conduction system. Therefore, while intentional under- or oversizing is a recognised strategy for limiting complications, unintentional (erroneous) prosthesis–annular mismatch can result in significant harm.

The modality that is most widely used now worldwide to perform pre-procedural sizing of the annulus is MDCT, and the most recent American College of Cardiology expert consensus statement on TAVR [17••] advises that TEE is unnecessarily invasive in the high-risk, frail TAVR population and should not be used routinely pre-TAVR. However, there is ample data that pre-procedural TEE offers benefits beyond valve sizing that merit its continued use. We have performed over 1000 pre-TAVR TEE studies in our centre without any complications.

Moreover, the actual imaging modality used is of less importance than the expertise of the imaging staff; consequently, it is best to use the modality with which a given centre has most expertise. Because patients with renal insufficiency may have a contraindication to contrast required for MDCT, and those with oesophageal pathology may not be candidates for TEE, it is important to have both MDCT and 3D TEE imaging available within a TAVR centre.

## Beyond Sizing

The relative merits of TEE and MDCT within the sizing arena are debatable, depending on which bias the operator has formed. However, the sizing of the annulus is only one aspect of the imaging requirement of TAVR and it is in areas beyond this that the modalities finally separate into discrete strengths: echocardiography for its unparalleled capacity to assess functional heart status and MDCT for its delineation of significant arterial routes.

Over the last decade, considerable data on TAVR procedures have accumulated. Certain pre-procedural characteristics are potentially disadvantageous when selecting patients for TAVR. Many of these are echocardiographic, for instance, left ventricular systolic or diastolic dysfunction, severe mitral valve disease or severe pulmonary hypertension may each increase the procedural risk or limit its benefit [17••]. Recently, pre-procedural baseline diastolic dysfunction and the absence of reverse remodelling after TAVR have both been shown to be negatively associated with 1-year mortality [18, 19]. In addition, mitral regurgitation (MR) is an important predictor of TAVR outcome, with secondary MR likely to improve post-procedure while primary MR typically does not [17••], an important consideration in patient selection and risk stratification. There is also now increasing awareness of tricuspid regurgitation as a potential adverse indicator, with recent work demonstrating pre-procedural tricuspid annular diameter as predictive of significant tricuspid regurgitation at 1 year [20]. With respect to the composition of the annulus and surrounding structures, TEE offers identification of the extent and location of calcification which may interfere with valve deployment. For instance, significant asymmetrical calcium deposition in the LVOT can result in paravalvular leak post-procedurally due to incomplete apposition of the valve struts against the surrounding wall. In addition, it is also a well-documented risk factor for iatrogenic ventricular septal defect formation.

These issues relate predominantly to those that can be identified by TEE, however, echocardiography can also be an important tool in identifying the functional response of a stenotic aortic valve. One potentially unfavourable echocardiographic feature pre-TAVR which is attracting significant recent interest is that of low-gradient aortic stenosis, present in up to 40% of aortic stenosis patients [21] and known to carry a poorer TAVR outcome [22]. Low-gradient aortic stenosis is of three subtypes depending on the mechanism involved: low-flow low-gradient (LFLG), paradoxical LFLG and normal-flow low-gradient [21, 22]. Paradoxical LFLG aortic stenosis is often seen in those with a small LV cavity resulting in a reduction in stroke volume despite the preserved EF, while the latter category of normal-flow low-gradient is thought secondary to elevated afterload such as systemic hypertension, driving down the gradient across the valve. The potential importance of low-gradient disease is currently being investigated in the TAVR UNLOAD study, a randomised controlled trial assessing optimal medical therapy versus optimal medical therapy plus TAVR in those with reduced left ventricular systolic function and moderate aortic stenosis [23]. Stress echocardiography is unrivalled for valve interrogation in LFLG aortic stenosis, and it is only when it is unfeasible for a patient to undergo stress testing that assessment by other means should be considered. In these scenarios, MDCT has been gaining traction for the estimation of the degree of aortic valve calcification as a pseudo-marker of low-gradient disease when stress testing is not feasible. The aortic valve calcium score derived by MDCT correlates strongly with the severity of stenosis, rate of progression and clinical outcomes [24]. Less impressive has been the use of MRI velocity encoded flow imaging which will consistently underestimate aortic velocities and is therefore not useful in this setting [17••].

Therefore, the judicious use of stress echocardiography in the evaluation of discrepancies between the visual appearance of the valve and the Doppler estimation of severity is of enormous importance in identifying those who may still benefit from TAVR in spite of low resting gradients. Overall, in the functional assessment of valvular heart disease and in the identification of potentially important adverse predictors of outcome, echocardiography remains an indispensable tool in the selection of appropriate patients for TAVR quite apart from its use in the sizing arena.

## Vascular Access Planning

**Fig. 3:**
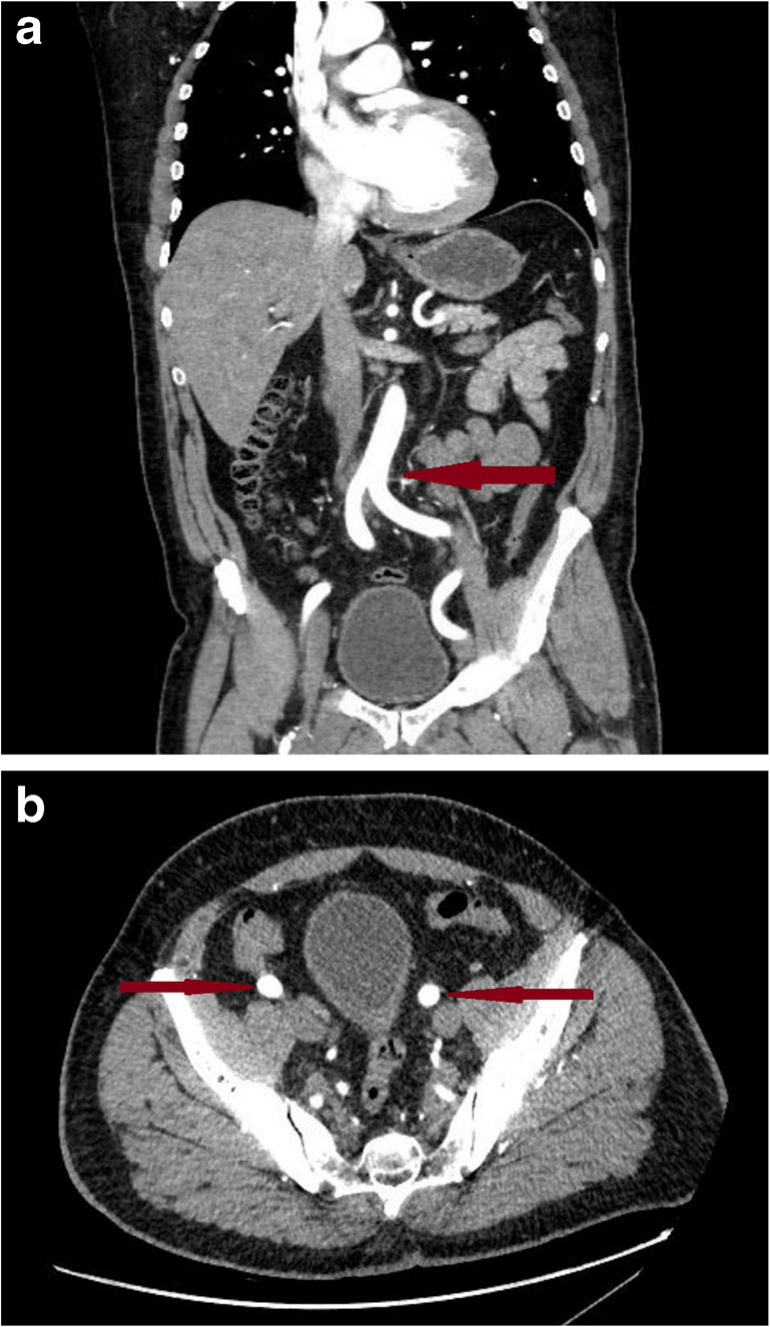
Coronal (**a**) and axial (**b**) views of the truncal vasculature. The *arrows* point to the aortofemoral bifurcation in the coronal image and the left and right femoral arteries in the axial image

It is important to assess the aorta and the peripheral arterial system that may be used during TAVR, and MDCT is ideal for this purpose. The rate of complications related to vascular access during TAVR has been reported as anything between 6.3 and 30.7% [25]. MDCT offers a virtual roadmap of the truncal vasculature allowing identification of vessel size, tortuosity, calcification and minimal lumen (Fig. 3a, b). Such information allows the planning of access routes with a view to minimising vascular complications. Significant tortuosity in the ileofemoral system increases the risk of trauma with the catheter system used during TAVR. Eccentric calcification is associated with easier access than vessels with circumferential calcification. Therefore, it is universally recommended to obtain vascular planning via MDCT pre-TAVR, with current recommendations advising a scanner with at least 64 detectors and a spatial resolution of 0.5–0.6 mm together with analysis on a dedicated workstation offering 3D manipulation [17••]. Gated sequencing is used for annular sizing, calcification burden, leaflet morphology and coronary assessment, while ungated imaging is sufficient for non-cardiac structures.

## MDCT and Coronary Angiography

With the evolution in MDCT and its pre-requisite use in TAVR planning, it has the potential to obviate the need for pre-procedural coronary angiography for assessment of the epicardial coronary arteries. The largest study involving over 300 participants did demonstrate reasonably good levels of sensitivity and specificity, prompting the conclusion that MDCT allows exclusion of significant coronary disease including in-stent restenosis [26]. However, other evidence suggests that while CT coronary angiography (CTCA) might allow detection of significant stenosis in proximal or mid-vessel segments, the specificity and positive predictive values are limited (73 and 72%, respectively) [27]. A recent study found diagnostic quality CTCA only in the left main stem (LMS) in a majority of patients and non-diagnostic CTCA rates ranging between 25 and 72% depending on the arterial segment analysed [28]. This study concluded that CTCA did not provide adequate diagnostic information to exclude severe coronary artery disease, primarily as a result of the extensive calcification present in the TAVR population. However, there is agreement that MDCT is useful in the evaluation of coronary bypass grafts [29]. Certainly, for the routine inspection of the native coronary system pre-TAVR, it seems that beam hardening artefact from extensive calcification will limit the applicability of the technique. The sensitivity and specificity could change if CT based fractional flow reserve (FFR) imaging becomes well validated in the future in the assessment of significant coronary artery disease. At present, there are still technical limitations in CT FFR that prevent its use much beyond the research sector [30]. Therefore, currently, CTCA is not yet proven sufficiently accurate in detecting important coronary artery disease in the TAVR population.

## Peri-Procedural Imaging

**Fig. 4:**
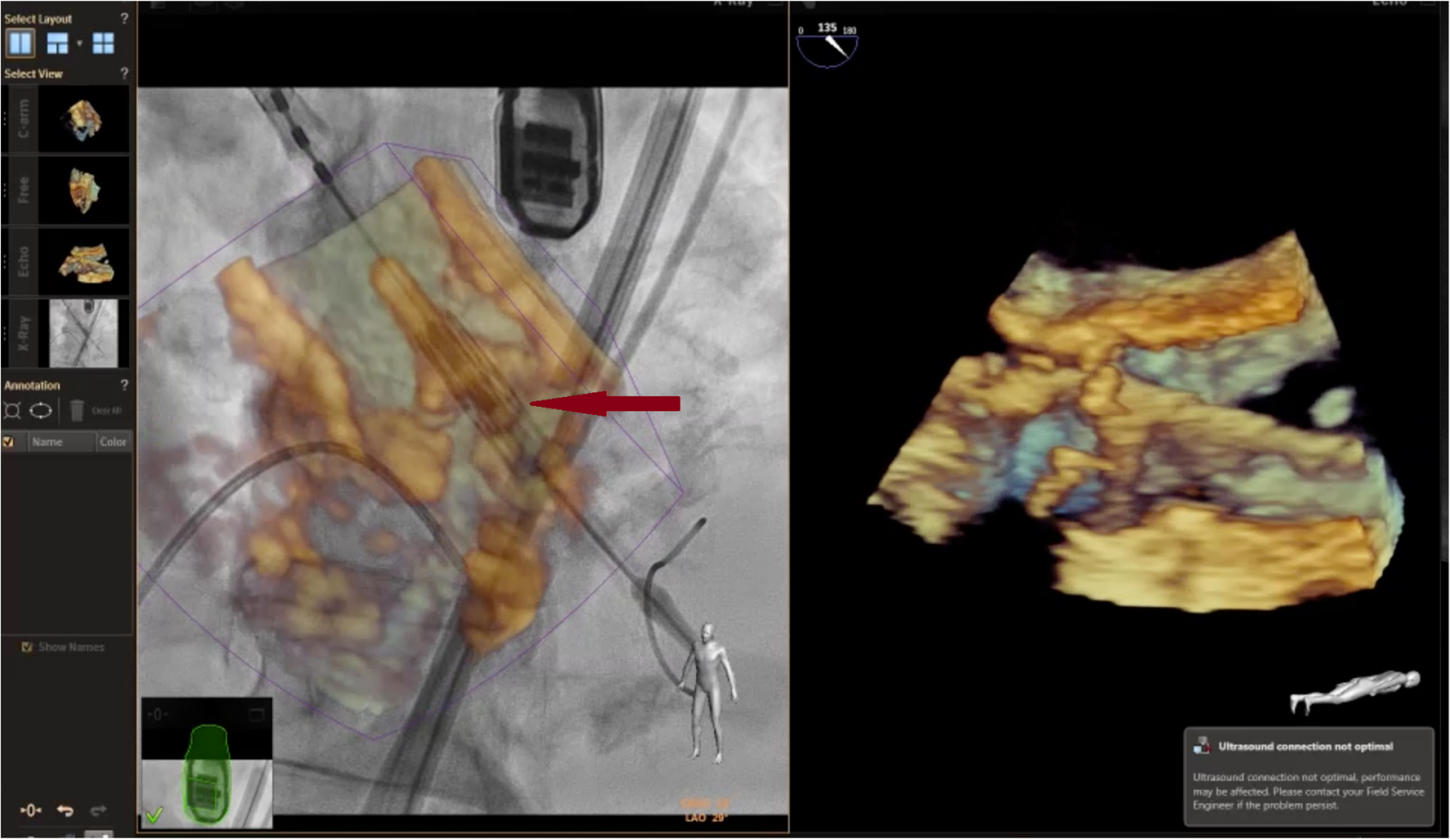
The EchoNavigator™ system allows real-time fusion of fluoroscopy (left side) and TEE (right side) images allowing the operator to see the aortic apparatus in 3D for optimal valve positioning. The arrow points to the pre-deployment Edwards Sapien™ valve seen within the 3D arrangement of the aortic annulus. The TEE probe seen just above is positioned cranially so as not to interfere with fluoroscopic imaging

Having integrated all available imaging modalities for the meticulous selection and preparation of patients for TAVR, the procedure itself is increasingly being performed under conscious sedation with fluoroscopic guidance. The shift away from general anaesthesia has led to fewer patients having TEE-guided TAVR, but this did not have a detrimental impact on outcomes, as reported in the French registries [31••]. This is likely in part a result of increasing operator experience. When TEE is used during implantation, it offers real-time diagnosis of complications such as tamponade, coronary occlusion, aortic dissection, aortic regurgitation and mitral regurgitation [25]. In addition, EchoNavigator™ technology provides fusion imaging of TEE and fluoroscopy in real time during TAVR, guiding the implanter to the most appropriate deployment position (Fig. 4). This makes zero contrast TAVR procedures possible, which can be advantageous in patients with renal impairment. However, in spite of the advantages offered by peri-procedural TEE, it is gradually being used less with many centres increasingly relying on TTE to detect peri-procedure complications and assess aortic regurgitation post-implant.

MDCT has not been adopted into the peri-procedural domain despite having shown promise as a contrast sparing tool by predicting fluoroscopic views. The feasibility of CT fusion imaging has been established in interventional procedures such as TAVR, paravalvular leak closure and in pulmonary vein anatomy for ablation studies [32]. The idea is to fuse or overlay reconstructed CT images onto fluoroscopic imaging in order to overcome the limitations common to all 2D modalities. This is not, however, a straightforward or timely process. Cone beam CT (CBCT) acquisition relies on rotation of the conventional coronary angiography C-arm in order to acquire a reconstructable 3D dataset. The problem with this is that the C-arm circles the patient much more slowly than with conventional gantry rotation and consequently an acquisition sequence may take up to 20 s with inevitable loss of temporal resolution [33]. Following acquisition, it is then necessary to reconstruct the required 3D datasets, a process that itself is comparatively time-consuming during live TAVR. Additionally, beyond these technical aspects of real-time acquisition and analysis, there remain concerns surrounding the additional radiation, although it appears that any added exposure is perhaps not as high as might be expected, reported in the region of 3.5% of total skin dose and 9.1% of total dose area product (DAP) [32]. Therefore, in recognition of each of the above issues, MDCT does not presently play a significant role in peri-procedural TAVR imaging.

## Current Status of Imaging for TAVR

Currently, interventionalists are using conscious sedation and transthoracic echocardiography for TAVR. Recently published registry data show diminishing numbers of TEE-guided procedures, falling from 60.7% in the earlier TAVI 2 registry to 32.3% in the later FRANCE TAVR data [31••]. Both in-hospital and 30-day mortality fell from 8.2% and 10.1% to 4.4% and 5.4%, respectively. Complication rates remained stable aside from a significant rise in permanent pacemaker implantation (up from 12.6 to 17.5%) and tamponade (up from 1.3 to 2%). Despite less procedures performed under general anaesthesia, there was a lower incidence of moderate or severe paravalvular leak in FRANCE TAVI, potentially attributable to increasing operator experience, refined annulus sizing, the availability of devices with a sealing skirt and the inclusion of lower risk profile patients in TAVR procedures. Therefore, it appears that the current TAVR landscape consists of more experienced operators implanting well-selected valves with generally fewer complications and better long-term outcomes.

## Conclusions

The development of TAVR has not only resulted in implantable AVR technology but has also given birth to new approaches to guide and image percutaneous procedures. These include real-time guidance pre-acquired roadmaps and intense planning. The available imaging modalities have each been evaluated and optimised to help predict accurately both the size of the intended implant and potential obstacles to procedural success. TEE and MDCT have been shown to be equitable in sizing the annulus appropriately. However, as many TAVR patients have potentially confounding structural or functional heart disease, it is likely that such patients may require both MDCT and TEE for optimal TAVR assessment and placement.
